# Association of the Hepatitis B Virus Large Surface Protein with Viral Infectivity and Endoplasmic Reticulum Stress-mediated Liver Carcinogenesis

**DOI:** 10.3390/cells9092052

**Published:** 2020-09-08

**Authors:** Wei-Ling Lin, Jui-Hsiang Hung, Wenya Huang

**Affiliations:** 1 Laboratory of Clinical Biochemistry, Department of Pathology, National Cheng Kung University Hospital, 138 Sheng Li Rd, Tainan 704, Taiwan; i34911060@gs.ncku.edu.tw; 2 Department of Biotechnology, Chia Nan University of Pharmacy & Science, 60 Sec. 1, Erren Rd, Rende Dist., Tainan 717, Taiwan; hung86@mail.cnu.edu.tw; 3 Department of Medical Laboratory Science and Biotechnology, College of Medicine, National Cheng Kung University, 1 University Rd, Tainan 701, Taiwan

**Keywords:** hepatitis B virus, large surface protein, endoplasmic reticulum, viral entry, sodium taurocholate cotransporting polypeptide, ground glass hepatocyte, pre-S deletion, hepatocellular carcinoma

## Abstract

Hepatitis B is the most prevalent viral hepatitis worldwide, affecting approximately one-third of the world’s population. Among HBV factors, the surface protein is the most sensitive biomarker for viral infection, given that it is expressed at high levels in all viral infection phases. The large HBV surface protein (LHBs) contains the integral pre-S1 domain, which binds to the HBV receptor sodium taurocholate co transporting polypeptide on the hepatocyte to facilitate viral entry. The accumulation of viral LHBs and its prevalent pre-S mutants in chronic HBV carriers triggers a sustained endoplasmic reticulum (ER) overload response, leading to ER stress-mediated cell proliferation, metabolic switching and genomic instability, which are associated with pro-oncogenic effects. Ground glass hepatocytes identified in HBV-related hepatocellular carcinoma (HCC) patients harbor pre-S deletion variants that largely accumulate in the ER lumen due to mutation-induced protein misfolding and are associated with increased risks of cancer recurrence and metastasis. Moreover, in contrast to the major HBs, which is decreased in tumors to a greater extent than it is in peritumorous regions, LHBs is continuously expressed during tumorigenesis, indicating that LHBs serves as a promising biomarker for HCC in people with CHB. Continuing efforts to delineate the molecular mechanisms by which LHBs regulates pathological changes in CHB patients are important for establishing a correlation between LHBs biomarkers and HCC development.

## 1. Introduction

Chronic hepatitis B (CHB) virus infection is the most important cause of hepatocellular carcinoma (HCC) worldwide. To date, one-third of the world’s population, which accounts for approximately 2 billion people, has been infected with HBV, and more than 200 million people suffer from CHB [1]. Individuals with CHB are at a greater than 100-fold increased risk of developing advanced liver diseases, including cirrhosis and HCC [2,3]. Therefore, HCC remains among the leading causes of cancer death worldwide. Effective prevention and control of HBV infection are very important public health issues in countries with epidemic HBV. In some regions, such as South Korea, Singapore and Taiwan, where the nationwide HBV vaccination of newborns has been executed for a few decades, incidences of HCC, and especially early-onset and childhood HBV-induced HCC, have been greatly reduced [4,5,6].

Hepatitis B virus was among the earliest identified viruses. It was discovered in 1965 by Dr. Baruch Blumberg and was originally designated the “Australia antigen” (later known as the HBV surface antigen) because it was found in a blood sample from an aboriginal Australian [7]. HBV is a member of the *Hepadnaviridae* family and is a small enveloped animal DNA virus. It is ~42 nm in diameter and enveloped by an ~34 nm icosahedral capsid [8]. Despite being a DNA virus, HBV replicates through the action of a viral reverse transcriptase to convert the pregenomic (pg) RNA intermediate into the viral DNA genome, which endows HBV with some retrovirus-like characteristics [9,10].

## 2. HBV Surface Proteins

**Figure 1:**
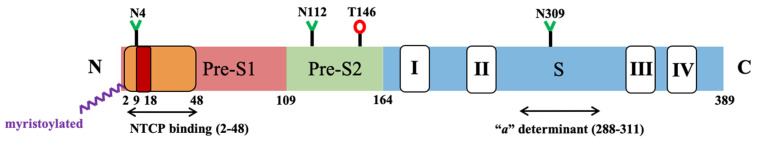
Composition of the HBV large surface protein. pre-S2, and S regions. Depending on the genotype, the pre-S1 region is in the size of 108, 109, or 119 amino acid (aa) residues. This graph is exemplified by the genotype D, where the pre-S1 region contains 108 aa. The pre-S2 and S regions contain 55 and 226 aa, respectively. The domain aa 2 to 48 in the pre-S1 region binds to NTCP to facilitate viral entry, where the aa 9–18 is the essential motif for the binding. The Gly2 residue is myristoylated, which is also important for the LHBs binding to NTCP. The S region contains 4 TM domains (I, II, II, and IV). The region aa 288–311 is the “*a*” determinant region, which is essential for induction of a protective humoral immune response by infection and vaccination. The common glycosylation sites on the protein include N-glycosylation at N4, N112, and N309, and O-glycosylation at T146.

The HBV surface gene contains three in-frame start codons that divide the gene into pre-S1, pre-S2, and S regions. Using multiple start codons, the HBV surface gene encodes three different proteins: large, medium, and small (i.e., major) surface proteins, all of which are indispensable components of the viral envelope [11]. The small surface protein is composed of the S region and mainly contains four transmembrane (TM) domains, which ensure that the HBs proteins are localized in virions and subviral particles (SVPs) at the membrane interface [12]. Compared with the small surface protein, the medium and large surface proteins contain additional N-terminal pre-S2 and pre-S1 and S2 regions, respectively [13]. The N-terminal pre-S regions have unique protein conformations that protrude from the endoplasmic reticulum (ER) membrane and lead to specific interplay between viral medium/large surface and host proteins, in addition to their roles as integral proteins in the virus envelope [14]. Glycosylation reactions have been documented to regulate the protein topologies of surface proteins, which are mainly incorporated across the cell and ER membranes [15] (Figure 1).

The HBV surface protein is a very sensitive biomarker for viral infection, given that it is expressed at high levels in all viral infection phases [16]. In addition to its location in the viral envelope, the surface protein is secreted outside cells through vesicle-mediated exocytosis to form filamentous and spherical SVPs that lack a genome-containing capsid; therefore, the HBV surface protein is a relatively stable biomarker indicating an infection history [17]. The viral surface protein also serves as a major epitope recognized by T cells, inducing T cell exhaustion through chronic stimulation and leading to failure of the immune surveillance machinery to recognize the virus [18,19]. To date, the molecular mechanisms by which the HBV surface protein triggers the disruption of the host immune response and by which it, in abundance, affects cellular functions through crosstalk with host factors, remain to be delineated.

## 3. The Pre-S1 Domain of the HBV Large Surface Protein in Viral Entry

The HBV large surface (LHBs) protein is essential for viral entry into the host cell. HBV initially binds to heparin sulfate proteoglycans (HSPGs) with low affinity [20,21]. The N-terminal amino acids in the pre-S1 region of the protein then bind tightly to the cell surface receptor sodium taurocholate co-transporting polypeptide (NTCP) to mediate viral entry through endocytosis [22]. It was reported that neutralizing antibodies targeting the pre-S1 region effectively prevented HBV entry; however, antibodies targeting the pre-S2 region had no effect on HBV infectivity [13]. A study by Yan et al. [22] reported that the overexpression of NTCP in human HepG2 cells renders these cells susceptible to HBV infection. The stably transfected NTCP-expressing HepG2 cell line has provided a much-needed and easily accessible platform for studying the virus and has also been used to identify chemicals targeting key steps of the virus life cycle, including cccDNA synthesis, and for the development of novel antivirals against infection [23].

The pre-S1 region of the LHBs protein is myristoylated at the Gly2 residue, which is dispensable for HBV virion formation but essential for infectivity, and it is also the major region targeted by neutralizing antibodies to inhibit virion binding to NTCP on hepatocytes [24,25]. In previous studies, it was found that the myristoylated pre-S1 peptide 2–48, but not the unmodified peptide, interacted with NTCP, probably at residues 157–165 of the NTCP protein [22,26]. Residues in the human NTCP (hNTCP) molecule critical for bile salt transport were also essential for NTCP to serve as an HBV receptor, as the substrates of NTCP inhibited pre-S1 lipopeptide binding to NTCP and viral infection [26]. Mutant hNTCP that is defective in substrate binding reduced the extent of HBV infection. The S267F variant of hNTCP, a common single-nucleotide polymorphism (SNP) found in East and Southeast Asian populations, has been found to render the protein defective in taurocholate-transporting activity and binding to HBV [27,28,29]. Similarly, the myristoylated pre-S1 peptide was reported to inhibit the transport of taurocholate into NTCP-expressing HepG2 cells [30]. Collectively, the molecular determinants critical for HBV entry overlap with those for bile salt uptake by NTCP, suggesting that viral infection may interfere with the normal function of NTCP.

## 4. Glycosylations of the LHBs Pre-S Regions that Regulate Protein Topology

Posttranslational modification (PTM) of the pre-S and S regions of HBV surface proteins plays key roles in regulating the topologies of cell membrane surface proteins and protein secretion [31]. The co-translational N-glycosylation modification of the L, M, and small HBs (SHBs) happens in the ER lumen and facilitates protein folding, stability, sorting, degradation, secretion, and immune response modulation [32]. These reactions are catalyzed by the oligosaccharyltransferase complex, which transfers N-linked glycans to the asparagine residue in the Asn-X-Thr/Ser sequence [33].

The S protein region, approximately 245 amino acid residues in size and with three or four TM domains, is first embedded into the ER membrane through co-translational translocation [34]. On the viral envelope, the inter-TM spacers are antigenic loops with unique conformations [35]. The major serological determinants for HBsAg subtypes are at positions 122 and 160, in the antigenic loop spacer between TM2 and TM3. Mutations at these and some other residues in this protein region have been associated with viral immune escape and are critical for vaccine and diagnostic escape phenomena, revealing their critical effects for HBs antigenicity [18,36]. Moreover, to facilitate the viral entry, the (+)-charged Arg122 and Lys141 residues interact with the (−)-charged HSPGs on the cell surface [37].

In the pre-S region, the Asn112 residue in the pre-S2 N-terminal region is linked with N-glycans to facilitate the assembly and secretion of virions and SVPs [38,39]. The first few amino acids at the pre-S2 N-terminal region are essential for viral export and infectivity, indicating that these N-glycosylation reactions modulate the L and medium HBs (MHBs) conformation, leading to maturation of viral and sub-viral particles [13]. However, the molecular mechanism controlling these N-glycosylation events in the viral life cycle remains unclear. Moreover, the Thr146 residue in the pre-S2 C-terminal region is partially modified by O-glycosylation, mainly in the Golgi apparatus for most HBV genotypes [38]. Intriguingly, Lambert et al. [32] demonstrated that LHBs undergoes co- and posttranslational N-glycosylation, not merely co-translational N-glycosylation as found for MHBs and SHBs. An analysis of the microsome-associated L chains revealed that Asn4 and Asn112 in the pre-S1 and pre-S2 domains, respectively, were modified with N-glycan [32]. Host cell-mediated N-glycosylation on L, but not on M or SHBs proteins is vital for viral morphogenesis [40]. Moreover, recent studies have shown that the N-glycans on LHBs are associated with ER stress-mediated cell cycle dysregulation and cell proliferation, triggering carcinogenic processes [41,42]. These findings clearly demonstrate that posttranslational modifications of LHBs play significant roles in regulating HBV carcinogenesis.

## 5. LHBs-induced Activation of Endoplasmic Reticulum Stress and Associated Carcinogenic Signaling Pathways

HBV surface proteins have been documented to associate with ER stress signaling pathways [43]. Highly expressed in HBV(+) hepatocytes, the co-translocated translational products of surface proteins reside in the ER for proper folding and posttranslational modifications, such as glycosylation, which plays an important role in the topology of the surface proteins [11]. The activation of ER stress by HBV HBx, LHB and SHB proteins has been identified in many reports [44,45,46,47,48]. HBx was found to inhibit apoptosis and cell cycle arrest by modulating ER stress response [44,48]. Cumulative overwhelming surface protein expression in the infected hepatocytes also causes ER overload and contributes to the pathological finding of ground glass hepatocytes (GGHs), which are characterized by the “foggy” appearance of the cytoplasm in hematoxylin and eosin-stained cells [46,49,50]. An analysis of CHB patients revealed that the overexpression of both LHBs and its pre-S mutants activates ER stress signaling pathways, which are associated with advanced liver disease and HCC development [46,51]. Rapid viral protein production after infection induces ER stress in host cells. For example, the cytomegalovirus ER-resident glycoprotein UL148 induces the unfolded protein response (UPR), leading to the activation of the two UPR downstream molecules the inositol-requiring enzyme-1 (IRE1) and the protein kinase R (PKR)-like ER kinase (PERK), which promote the expression of chaperone proteins for proper protein folding [52]. Moreover, HCV infection is associated with ER stress-mediated metabolic disorders such as insulin resistance caused by glucose homeostasis disturbance in cyclic AMP (cAMP)-responsive element-binding protein (CREB) phosphorylation-induced peroxisome proliferator-activated receptor gamma coactivator-1α (PGC-1α) activation, which regulates the glucose metabolic pathway [53].

The ER is involved in protein folding, modification and secretion. As the major organelle for protein posttranslational modification, a polypeptide translated in the ribosome directly enters the ER, where it is glycosylated and guided through modification steps to generate its final conformation [54]. This protein is then transported from the ER to the Golgi apparatus for final modifications. The unfolded protein response (UPR), activated in response to an accumulation of unfolded or misfolded proteins in the ER lumen, is a cellular stress response related to ER stress [55]. The UPR has three main functions: initially, it restores normal cell function by halting protein translation, degrading the misfolded protein, and activating the signaling pathways that lead to increased production of the molecular chaperones involved in protein folding [56]. Where misfolded proteins continually breach ER quality control, the UPR triggers apoptosis or chaperone proteins to facilitate the ER-associated protein degradation (ERAD) pathway, which is involved in protein retro-translocation from the ER to the cytosolic 26S proteasome for degradation [57]. 

ER stress, induced by prolonged and overwhelming protein production/misfolding, triggers a number of signaling pathways, including the UPR, the ER overload response (EOR), and steroid uptake pathways [55]. In the UPR, the chaperone glucose-regulated protein 78 (GRP78) is recruited to misfolded proteins, which results in its dissociation from the transmembrane receptor proteins PERK, IRE1 and ATF6 [58]. Such dissociation results in the receptor homodimerization and oligomerization indicative of an active state. Activated PERK phosphorylates eIF2α, inhibiting translation and resulting in cell cycle arrest. Meanwhile, the activated IRE1 cleaves its substrate, X-box-binding protein (XBP) 1, facilitating its conversion to its active form as transcription factor XBP1. Additionally, activated ATF6 is translocated to the Golgi apparatus, where it is cleaved by proteases to form an active fragment, which together with XBP1 binds to ER stress element (ERSE) promoters in the nucleus to promote the expression of proteins involved in the UPR. Additionally, in response to ER stress, the ER overload response triggers Ca^2+^ efflux from the ER lumen and reactive oxygen ion (ROI) release from mitochondria, resulting in oxidative stress and NF-kB nuclear localization and activation [58,59].

LHBs and its pre-S mutants trigger the activation of ER stress-associated signaling pathways [46,51,59]. In addition to the overwhelming production of viral protein in the ER, the mutation-induced protein misfolding of pre-S mutants, which is prevalent in chronic carriers, induces strong and sustained ER stress. Exogenous LHBs induces the phosphorylation of the ER stress molecule GRP78, the C/EBP homologous protein (CHOP) transcription factor, PERK and eIF2α, and promotes XBP1 splicing in liver cells [46,51,59]. In addition, the expression of LHBs in human HCC tissues has been correlated with the upregulation of the PKCα/Raf1/Src/PI3K/Akt signaling pathway, which is associated with ER stress [60]. A study by Hung et al. [59] reported that, similar to the ER stress inducers tunicamycin and thapsigargin, LHBs and pre-S mutants induce cyclooxygenase-2 (COX-2) activation through the p38 and NF-kB signaling pathway in an in vivo HCC model. In addition, LHBs also induces the ER stress-mediated EOR pathway, leading to Ca^2+^ efflux and ROS generation, which leads to oxidative DNA damage and genomic instability [51]. Moreover, the naturally occurring LHBs and MHBs pre-S deletions presented with immune escape and reduced secretion of virions, indicating the accumulation of the surface proteins in ER and Golgi apparatus [61,62]. Together, the accumulation of LHBs and its pre-S variants in the ER induces ER stress-related signaling pathways, which promote cell survival and carcinogenesis. The abrogation of LHBs-induced ER stress signaling pathways is a promising prophylactic therapy against HBV-related HCC.

## 6. ER Stress-mediated Metabolic Pathways Associated with LHBs

The metabolic changes in chronic HBV patients are reportedly correlated. A study analyzed the serum from patients of chronic HBV infection at various clinical phases and found that the metabolic status or pathways of carbohydrate, protein and lipid is altered in the immune-tolerance phase of chronic HBV in patients [63]. ER stress signaling has been linked to metabolic homeostasis in many aspects [64]. Upon ER stress, the XBP1 branch regulates glucose metabolism through the transcription factor hypoxia inducible factor 1α (HIF1α) and glucose transporter (GLUT) 1 and 2 [65,66,67]. The PERK/ATF4 branch is critically involved in the regulation of amino acid biosynthesis, mitochondrial stress responses and glycolysis [68,69,70]. Through the regulation of ER homeostasis and metabolic pathways, the UPR mediates cell adaptation to stress conditions and determines cell fate and function. Interestingly, LHBs pre-S variants interact with acid α-glucosidase, which plays an important role in the alternation of the glycogen balance [65]. The HCC tissues in transgenic mice carrying the LHBs pre-S variants and humans with HCC also presented with glycogen depletion, consistent with the notion that LHBs disrupts ER stress-mediated cellular glucose homeostasis [71]. Moreover, a study by Teng et al. [72] reported that LHBs activated the oncogenic factor mTOR through the induction of ER stress-dependent vascular endothelial growth factor A (VEGFA)/AKT. The LHBs pre-S2 variant was found to initiate a mTOR-dependent glycolytic pathway involving eukaryotic translation initiation factors, Yin Yang 1 (YY1), MYC, and the solute carrier family 2 member 1 (SLC2A1), resulting in aberrant glucose uptake and lactate production in tumorigenic processes [71].

On the other hand, prolonged UPR activation has been reported to cause a decrease in protein synthesis and glycolysis as the cell restores proteostasis and glucose homeostasis in the ER [73]. Thus, it also results in decreased O-GlcNAc posttranslational modification, which is in line with reduced glucose metabolism. It was found that metabolic decreases were prevented when the UPR/IRE1 pathway was inhibited, indicating that the activation of IRE1 signaling induced a reduction in glucose metabolism as part of an adaptive response [73]. Other studies also showed that, upon chronic ER stress or in obesity, the ATF6 branch is inactivated, resulting in increased gluconeogenesis [74]. In addition, PERK-eIF2α signaling positively impacts gluconeogenesis through the activation of CCAAT-enhancer-binding protein α/β (C/EBPα/β) transcription factors [75]. Collectively, hepatocytes with long-term chronic HBV infection are likely to manifest the aberrant ER stress-mediated metabolic homeostasis that has been delineated from a global perspective.

HBV LHBs was also found to cause lipid accumulation induced through the glycogen synthase kinase (GSK)-3β/acyl-CoA synthetase (ACSL) 3 signaling pathway, and triacsin C and GSK-3β inhibitors abrogated this effect [76]. Therefore, lipid accumulation may be caused by the ER stress-mediated GSK-3β signaling pathway, which enhances the expression of long-chain ACSL3 and consequently leads to lipid synthesis. In addition, in the HBV transgenic mouse model, activation of the mTOR signaling pathway causes the sterol regulatory element binding transcription factor 1 (SREBF1)-induced upregulation of ATP citrate lyase (ACLY) and fatty acid desaturase 2 (FADS2), leading to an increase in triglycerides and cholesterol [77]. These findings clearly indicate that LHBs-induced ER stress-mediated lipid metabolism disturbance is functionally linked to HBV tumorigenesis.

The effect of HBV on bile acid metabolism is based on the function of the HBV receptor NTCP, which is the key sodium/bile acid cotransporter and mediates bile acid transport from the portal vein into the liver [78]. Bile acids are synthesized from cholesterol and conjugated to glycine or taurine in the liver [79]. As a cotransporter, NTCP binds two sodium ions and one conjugated bile acid molecule, thereby driving a hepatic influx of bile salts [78]. Recent studies have indicated that HBV infection attenuates normal NTCP function during bile acid transport, and myrcludex B, a synthetic N-acetylated pre-S1 lipopeptide that disrupts pre-S1 binding to NTCP, inhibits NTCP binding to bile acid substrates [80]. Additionally, the results from a clinical trial of myrcludex B on HDV infection revealed that the treated patients showed a moderate elevation in taurine-conjugated and glycine-conjugated bile acids in their peripheral blood, suggesting that the association of NTCP with the LHBs pre-S1 region causes hepatocytes to exhibit deficient bile acid absorption and may potentially alter bile acid biosynthesis [81].

## 7. LHBs Pre-S Genetic Variations in Ground Glass Hepatocytes Contribute to the ER Stress-mediated Carcinogenic Processes

The HBV genome harbors a high degree of genetic variation in chronic carriers. According to a longitudinal study of DNA-sequencing analysis of samples from chronic carriers, the mean number of nucleotide substitutions/site/year in the HBV genome was estimated to be ~7.9 × 10^−5^, resulting in a rate of nonsynonymous mutations of approximately 2 × 10^−5^ amino acid replacements/site/year [82]. Such a high mutation rate is due mainly to insufficient proofreading of the viral polymerase, which creates high error rates in viral genome synthesis [83]. Other causes include the emergence of immune escape variants and drug-resistant clones that develop after long-term use of antiviral therapies [84]. Most of these selected viral variants are difficult to eradicate and are sustainable for years in host cells. Therefore, some variants are prevalent in chronic and advanced liver diseases such as cirrhosis and HCC. Moreover, these variants have been found to aberrantly interact with host proteins and trigger pathogenic changes through the virus–host interplay, leading to carcinogenesis [85]. The precore/core promoter mutations in HBV genotypes from A to J, which have distinct geographic distributions, have been recognized as being predictive of liver disease progression, including HCC [86]. In the case of the surface protein, the main cancer-related variations are in the pre-S1/S2 regions, where the nonmembrane-associated conformations are found, allowing unique viral-host protein interactions [87,88]. In addition, some pre-S variants have been identified as significant etiological factors for liver pathological changes in HBV carriers [45,49,89].

Pathologists first identified pre-S deletion variants in GGHs in HBV-related HCC in the early 1970s, when a GGH was indicated by the “foggy” and “glassy” appearance of its cytoplasm after HE staining due to surface protein accumulation in the intracellular or ER lumen [90]. After the introduction of immunohistochemistry in the 1980s, some studies demonstrated that different types of GGHs correlated to expression patterns of HBV surface antigens in chronic HBV infected cells. GGH types I and II were associated with partial deletions in the pre-S1 and pre-S2 regions, respectively [91]. Type I GGHs usually scatter singly in hepatic lobules where the surface protein is expressed in an “inclusion-like” pattern, whereas type II GGHs express the surface protein in a unique expression pattern at the cell margin and consistently in clustered nodules and are frequently associated with cirrhosis or HCC [49]. This suggests that type II GGHs are associated with clonal proliferation and carcinogenesis. A later study by Wang et al. [46] showed that GGHs harbor pre-S deletion variants that accumulate in the ER and induce ER stress signaling.

### 7.1. Pre-S1 Deletion Variants

A deletion in the pre-S1 region has been documented to be associated with liver pathology in chronic HBV infection. Fan et al. found that the C-terminal portion of the pre-S1 region is commonly deleted and does not affect viral entry, which is mediated through the N-terminal region [49,92]. Some clones have large deletions that span the pre-S1 and part of the pre-S2 regions, resulting in the loss of the pre-S2 start codon for MHBs translation. Whether the loss of the medium surface protein affects the viral life cycle remains to be clarified. Thus, the pre-S1 region, which has hydrophilic properties, controls the N-terminal topology of the protein [40,93]. A newly translated LHBs protein is embedded into the ER membrane with the N-terminal ends constituting approximately half of the protein protruding into the cytosol and the other half protruding into the ER lumen, leading to cross talk between viral and host factors in the ER and cytosol [93]. It has been reported that pre-S1 deletions cause the disorientation of the LHBs N-terminal end, triggering ER stress signaling and the associated cell apoptosis pathway [41]. Moreover, it was found that the LHBs pre-S1 deletion mutant exhibited compromised cell survival and colonization and therefore was not linked to pro-oncogenic activity [45].

### 7.2. Pre-S2 Deletion Variants

The HBs pre-S2 region, which starts at the ATG start codon of the medium surface protein, is relatively small with 55 amino acids. The common deletions in the pre-S2 region are in its N-terminus [92]. Shen et al. [94] found that, in the HBV acute infection phase, no pre-S2 deletion was prevalent; however, in the late phases of CHB and HCC, the deletion rates increased to approximately 40% in the serum, indicating that pre-S deletions emerge in the long-term infection period and are eventually selected, probably because they demonstrate pro-proliferative properties.

**Figure 2:**
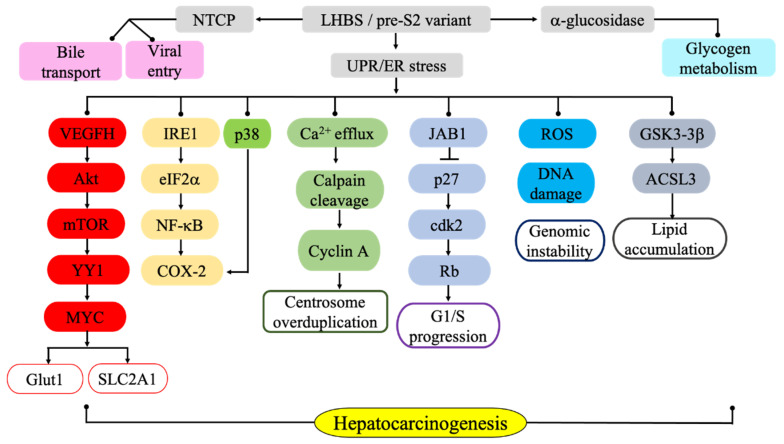
Involvement of the HBV large surface protein in viral entry, glycogen metabolism, and ER stress-associated carcinogenic pathways.

Previous pathological observations suggested that pre-S2 deletion variants are prevalent in type II GGHs, which are characterized by a marginal surface protein expression pattern in IHC analyses, indicating excessive surface protein accumulation in the cytosol [46,49]. The common in-frame deletions start at the 3rd or 4th amino acid in the pre-S2 region and span approximately 16–18 residues. Some mutant clones also contain point mutations at the pre-S2 start codon (ATG to ATA), resulting in the loss of MHBs function [49,94]. The intracellular accumulation of the pre-S2 mutant LHBs has been found to stimulate the formation of hepatic nodules, enhanced clonal expansion and induced carcinogenesis [46,51]. Hsieh et al. [51] reported that due to mutation-induced protein misfolding, the pre-S2 mutant protein accumulates in the ER and initiates ER stress-dependent DNA damage, centrosome overduplication, and genomic instability [95]. The expression of the pre-S2 mutant protein induced oxidative DNA damage, as demonstrated by an increase in 8-hydroxyguanosine on DNA lesions and the induction of oxidative DNA repair genes 8-oxoguanine glycosylase 1 (*ogg1*) and X-ray cross-complementation 1 (*xrcc1*) [51]. The pre-S2 mutant LHBs also induces ER stress-mediated VEGF/Akt/mTOR and NF-kB/COX-2 signaling pathways, triggering cell inflammation and transformation [59,96]. Moreover, the pre-S2 mutant protein specifically interacts with c-Jun activation domain binding protein 1 (JAB1), which enhances activator protein-1 transcriptional activity and cell proliferation. Through its binding to JAB1, the pre-S2 mutant protein induces JAB1 nuclear translocation, which activates p27/retinoblastoma/Cdk2/cyclin A pathways and leads to cell cycle progression [88]. It was also reported that transgenic mice carrying pre-S2 LHBs exhibit genomic instability, as indicated by the high levels of genome-wide copy number variations [87]. In some of these mice, HCC also developed. Taken together, these results suggest that pre-S2 mutant LHBs is an important viral oncoprotein that promotes liver carcinogenesis (Figure 2).

The pre-S1 region of the LHBs protein interacts with NTCP on the host cell membrane to mediate the viral entry and bile acid transport. The LHBs and its pre-S variants interact with acid α-glucosidase and regulates alteration of the glycogen balance. The pre-S mutation-induced protein misfolding induces unfolded protein response (UPR) and the ER stress-mediated signaling pathways, including IRE1/p38-mediated NF-kB and COX-2 activation, calcium efflux, calpain cleavage and cyclin A overexpression, which leads to centrosome over-duplication. The ER stress-dependent ROS production and DNA damage also occur and lead to genomic instability. In addition, LHBs and its pre-S2 variant induce the oncogenic factor mTOR through the induction of ER stress-dependent VEGFA and AKT activation, leading to the mTOR-dependent glycolytic pathway involving eIF2α, YY1, MYC, and SLC2A1, which results in aberrant glucose uptake and lactate production in tumorigenic processes. Moreover, the pre-S2 variant surface protein binds to JAB1 and induces JAB1 nuclear translocation, which activates p27/retinoblastoma/Cdk2/cyclin A pathways and leads to cell cycle progression. In addition, LHBs expression triggers lipid synthesis by the GSK-3β/ACSL3 signaling pathway, likely through the ER stress. Through activating multiple pro-oncogenic pathways, LHBs plays a significant role in HBV-related HCC development. 

## 8. LHBs in Infection Phases and HCC: Detection and Applications

To monitor the therapeutic efficacies of antiviral drugs in CHB individuals, it is essential to develop convenient biomarkers that can sensitively and rapidly reflect trends in viral reproductive activity. To date, the most reliable marker is the viral genome DNA level, which indicates viral replication activity. The most common CHB viral marker, HBsAg, also decreases with decreases in viral replication activity; however, due to its long half-life and high abundance in serum, the level of HBsAg changes much more slowly than the DNA level, even when antiviral therapy is highly effective [97]. Nevertheless, although the viral DNA titer is a sensitive early biomarker for determining antiviral therapeutic efficacy, the experimental procedures for its detection require DNA extraction and real-time polymerase chain reaction, which are relatively time-consuming and costly. Thus, some recent studies have proposed LHBs as a promising marker for HBV life cycle progression [98]. In contrast to SHBS, which is highly expressed in free form or spherical SVPs in serum and in the virion envelope, LHBs is mainly an integral component of virion envelope and some filamentous SVPs [99,100]. The LHBs pre-S1/S2 regions have been shown to play pivotal roles in the HBV life cycle in the following ways: (1) the N-terminal domain of the pre-S1 region directly binds to the receptor NTCP and mediates entry of the virus into the hepatocyte; (2) LHBs is essential for virus budding and assembly of the envelope proteins of the nucleocapsid. The N-terminal region of the pre-S1 domain interacts with the host cell membrane and facilitates exocytosis; and (3) the pre-S2 domain, which together with the small S domain constitutes the medium S protein, is essential for the secretion of the S protein and virus envelope assembly [11,101].

LHBs is also closely involved in cccDNA transcription because of its transactivation function [102,103]. LHBs has been shown to have a significantly higher correlation with the HBV DNA titer than does HBsAg in patients who receive nucleotide analog (NA) antiviral treatments [99]. The levels of both HBV DNA and LHBs decreased during antiviral treatment at very similar rates [104]. The continuous positive LHBs in patient sera predicted HBV DNA conversion, even for patients who continued to receive antiviral treatments [104]. It was also reported that early serum LHBs level was a strong predictor of a viral response to peginterferon alfa-2a and entecavir in HBeAg-positive CHB patients [105]. The predictive capability of the LHBs level 4 weeks after infection tended to be higher than that of HBsAg or HBV DNA level in a peginterferon alfa-2a group. Another study demonstrated that the level of LHBs efficiently reflected the replication status of the virus in patients with HBeAg-negative diseases, indicating that LHBs can potentially be used to monitor the effectiveness of antiviral treatments [104]. Given that the detection of serum LHBs is much more time- and cost-effective than that of HBV DNA by qPCR, LHBs may serve as a screening biomarker for antiviral therapy efficacy.

We recently found that, in contrast to that of small HBs, the expression of LHBs is maintained in HCC and likely plays an important role in promoting tumor progression [106]. Immunohistochemical staining was performed to examine the expression of various surface proteins in tumors surgically resected from HBV-related HCC patients. It was found that the major S protein was lower in the tumors than in the peritumorous samples, in which the HBs signals were usually intense, suggesting that the HBs gene promoter was downregulated in the process of carcinogenesis. However, in an IHC analysis using the pre-S1 region monoclonal antibody that specifically detected LHBs, the tumorous regions, similar to the nontumorous regions, presented with strong staining signals in more than 90% of the cases. These data clearly demonstrated that, in contrast to the major HBs, LHBs is continuously expressed in carcinogenesis and probably plays an important role in cancer progression. Moreover, independent of the American Joint Committee on Cancer (AJCC) tumor stage, the serum pre-S mutant level was positively correlated with the risk for HCC recurrence after hepatectomy. The results of a multivariate regression analysis show that the serum pre-S2 mutant levels at the AJCC tumor stage served as significant predictive high-risk biomarkers for HCC progression in patients who have undergone primary treatments, indicating that they had benefitted from the use of precision medicine in HCC therapy [106].

## 9. Conclusions

LHBs, containing the integral pre-S1 domain mediating the binding of the viral envelope to the receptor NTCP on hepatocytes, is essential for viral infection and life cycle. The expression of viral LHBs in chronic HBV carriers also triggers a sustained ER overload response, leading to the activation of ER stress signaling pathways. However, the exact role of LHBs in liver carcinogenesis has not been clearly demonstrated. It was reported that LHBs transgenic mice developed HCC. A recent study showing sustained expression of LHBs in human HCC tumors also suggested that LHBs, in contrast to SHBs, is uniquely associated with HBV-related carcinogenic processes. Although some clinical observations have been made to support a correlation of LHBs with viral replication, the molecular mechanisms by which LHBs plays a unique role in regulating the HBV life cycle and responses to antiviral therapies remain to be delineated.

## Abbreviations

ACSL	acyl-CoA synthetase
ACLY	ATP citrate lyase
AJCC	American Joint Committee on Cancer
C/EBPα/β	CCAAT-enhancer-binding protein α/β
CHOP	C/EBP homologous protein
CHB	Chronic hepatitis B
COX-2	Cyclooxygenase-2
CREB	cAMP-responsive element-binding
EOR	ER overload response
ER	Endoplasmic reticulum
ERAD	ER-associated protein degradation
ERSE	ER stress element
FADS2	Fatty acid desaturase 2
GGH	ground glass hepatocyte
GLUT	Glucose transporter
GSK	Glycogen synthase kinase
HBV	Hepatitis B virus
HIF-1	Hypoxia-inducible factor 1
HCC	hepatocellular carcinoma
JAB1	c-jun activation domain binding protein 1
LHBS	Large hepatitis B virus surface
MHBS	medium hepatitis B virus surface
NA	Nucleotide analog
NTCP	Sodium taurocholate cotransporting protein
OGG1	Oxoguanine glycosylase 1
PERK	Protein kinase R-like ER kinase
pg	Pregenomic
PTM	posttranslational modification
ROI	Reactive oxygen ion
ROS	Reactive oxygen species
SHBS	small hepatitis B virus surface
SNP	single nucleotide polymorphism
SLC2A1	Solute carrier family 2 member 1
SVP	Subviral particle
TM	transmembrane
UPR	unfolded protein response
VEGF	Vascular endothelial growth factor
XBP1	X-box-binding protein 1
XRCC1	X-ray cross-complementation 1
YY1	Yin Yang 1

## References

[1] World Health Organization (WHO) (2017). Global Hepatitis Report. https://apps.who.int/iris/bitstream/handle/10665/255016/9789241565455-eng.pdf.

[2] Arbuthnot P., Kew M. (2001). Hepatitis B virus and hepatocellular carcinoma. Int. J. Exp. Pathol..

[3] Inoue T., Tanaka Y. (2020). Novel biomarkers for the management of chronic hepatitis B. Clin. Mol. Hepatol..

[4] Chang M.H., Chen C.J., Lai M.S., Hsu H.M., Wu T.C., Kong M.S., Liang D.C., Shau W.Y., Chen D.S. (1997). Universal hepatitis B vaccination in Taiwan and the incidence of hepatocellular carcinoma in children. Taiwan Childhood Hepatoma Study Group. N. Engl. J. Med..

[5] Chang M.H., You S.L., Chen C.J., Liu C.J., Lee C.M., Lin S.M., Chu H.C., Wu T.C., Yang S.S., Kuo H.S. (2009). Taiwan Hepatoma Study Group. Decreased incidence of hepatocellular carcinoma in hepatitis B vaccinees: A 20-year follow-up study. J. Natl. Cancer Inst..

[6] Beasley J., Hwang L.Y., Lin C.C., Chien C.S. (1981). Hepatocellular carcinoma and HBV. A prospective study of 22,707 men in Taiwan. Lancet.

[7] Blumberg B.S., Alter H.J., Visnich S. (1965). A ‘‘new’’ antigen in leukemic sera. JAMA.

[8] Patient R., Hourioux C., Roingeard P. (2009). Morphogenesis of hepatitis B virus and its subviral envelope particles. Cell Microbiol..

[9] Zuckerman A.J., Baron S. (1996). Hepatitis viruses. Baron’s Medical Microbiology.

[10] Seeger C., Zoulin F., Mason W.S., Knipe D.M., Howley P.M. (2007). Hepadnaviruses. Field’s Virology.

[11] Churin Y., Roderfeld M., Roeb E. (2015). Hepatitis B virus large surface protein: Function and fame. Hepatobiliary Surg. Nutr..

[12] Siegler V.D., Bruss V. (2013). Role of transmembrane domains of hepatitis B virus small surface proteins in subviral-particle biogenesis. J. Virol..

[13] Le Seyec J., Chouteau P., Cannie I., Guguen-Guillouzo C., Gripon P. (1998). Role of the Pre-S2 Domain of the Large Envelope Protein in Hepatitis B Virus Assembly and Infectivity. J. Virol..

[14] Bruss V. (2007). Hepatitis B virus morphogenesis. World J. Gastroenterol..

[15] Dobrica M.-O., Lazar C., Branza-Nichita N. (2020). N-glycosylation and N-glycan processing in HBV biology and pathogenesis. Cells.

[16] Hadziyannis E., Laras A. (2018). Viral biomarkers in chronic HBeAg negative HBV infection. Genes.

[17] Hu J., Liu K. (2017). Complete and incomplete hepatitis B virus particles: Formation, function, and application. Viruses.

[18] Lazarevic I., Banko A., Miljanovic D., Cupic M. (2019). Immune-escape hepatitis B virus mutations associated with viral reactivation upon immunosuppression. Viruses.

[19] Heim K., Neumann-Haefelin C., Thimme R., Hofmann M. (2019). Heterogeneity of HBV-specific CD8^+^ T-cell failure: Implications for immunotherapy. Front. Immunol..

[20] Watashi K., Wakita T. (2015). Hepatitis B virus and hepatitis D virus entry, species specificity, and tissue tropism. Cold Spring Harb. Perspect. Med..

[21] Sumiya M., Liu Q., Yoshimoto N., Lijima M., Tatematsu K., Nakai T., Okajima T., Kuroki K., Ueda K., Kuroda S. (2016). Cellular uptake of hepatitis B virus envelope L particles is independent of sodium taurocholate cotransporting polypeptide, but dependent on heparan sulfate proteoglycan. Virology.

[22] Yan H., Zhong G., Xu G., He W., Jing Z., Gao Z., Huang Y., Qi Y., Peng B., Wang H. (2012). Sodium taurocholate cotransporting polypeptide is a functional receptor for human hepatitis B and D virus. eLife.

[23] Sun Y., Qi Y., Peng B., Li W. (2017). NTCP-reconstituted in vitro HBV infection system. Methods Mol. Biol..

[24] Falco S.D., Ruvo M., Verdoliva A., Scarallo A., Raimondo D., Raucci A., Fassina G. (2001). N-terminal myristylation of HBV preS1 domain enhances receptor recognition. J. Pept. Res..

[25] Glebe D., Urban S. (2007). Viral and cellular determinants involved in hepadnaviral entry. World J. Gastroenterol..

[26] Ni Y., Lempp F.A., Mehrle S., Nkongolo S., Kaufman C., Falth M., Stindt J., Koniger C., Nassal M., Kubitz R. (2014). Hepatitis B and D viruses exploit sodium taurocholate co-transporting polypeptide for species-specific entry into hepatocytes. Gastroenterology.

[27] Yan H., Peng B., Liu Y., Xu G., He W., Ren B., Jling Z., Sui J., Li W. (2014). Viral entry of hepatitis B and D viruses and bile salts transportation share common molecular determinants on sodium taurocholate cotransporting polypeptide. J. Virol..

[28] Yang F., Wu L., Xu W., Liu Y., Zhen L., Ning G., Song J., Jiao Q., Zheng Y., Chen T. (2019). Diverse effects of the NTCP p.Ser267Phe variant on disease progression during chronic HBV infection and on HBV preS1 variability. Front. Cell. Infect. Microbiol..

[29] Lee H.W., Park H.J., Jin B., Dezhbord M., Kim D.Y., Han K.-H., Ryu W.-S., Kim S., Ahn S.H. (2017). Effect of S267F variant of NTCP on the patients with chronic hepatitis B. Sci. Rep..

[30] König A., Döring B., Mohr C., Geipel A., Geyer J., Glebe D. (2014). Kinetics of the bile acid transporter and hepatitis B virus receptor Na+/taurocholate cotransporting polypeptide (NTCP) in hepatocytes. J. Hepatol..

[31] Yang F. (2018). Post-translational modification control of HBV biological processes. Front. Microbiol..

[32] Lambert C., Prange R. (2007). Posttranslational N-glycosylation of the hepatitis B virus large envelope protein. Virol. J..

[33] Hyakumura M., Walsh R., Thaysen-Andersen M., Kingston N.J., La M., Lu L., Lovrecz G., Packer N.H., Locarnini S., Netter H.J. (2015). Modification of asparagine-linked glycan density for the design of hepatitis B virus virus-like particles with enhanced immunogenicity. J. Virol..

[34] Suffner S., Gerstenberg N., Patra M., Ruibal P., Orabi A., Schindler M., Bruss V. (2018). Domains of the hepatitis B Virus Small Surface Protein S Mediating Oligomerization. J. Virol..

[35] Le S.I., Thedja M.D., Roni M., Muljono D.H. (2010). Prediction of conformational changes by single mutation in the hepatitis B virus surface antigen (HBsAg) identified in HBsAg-negative blood donors. Virol. J..

[36] Purdy M.A. (2007). Hepatitis B virus S gene escape mutants. Asian J. Transfus. Sci..

[37] Sureau C., Salisse J. (2013). A conformational heparan sulfate binding site essential to infectivity overlaps with the conserved hepatitis B virus a-determinant. Hepatology.

[38] Julithe R., Abou-Jaoudé G., Sureau C. (2014). Modification of the hepatitis B virus envelope protein glycosylation pattern interferes with secretion of viral particles, infectivity, and susceptibility to neutralizing antibodies. J. Virol..

[39] Cai X., Zheng W., Pan S., Zhang S., Xie Y., Guo H., Wang G., Li Z., Luo M. (2018). A virus-like particle of the hepatitis B virus preS antigen elicits robust neutralizing antibodies and T cell responses in mice. Antivir. Res..

[40] Pastor F., Herrscher C., Patient R., Eymieux S., Moreau A., Burlaud-Gaillard J., Seigneuret F., de Rocquigny H. (2019). Direct interaction between the hepatitis B virus core and envelope proteins analyzed in a cellular context. Sci. Rep..

[41] Choi Y.-M., Lee S.-Y., Kim B.-J. (2019). Naturally occurring hepatitis B virus mutations leading to endoplasmic reticulum stress and their contribution to the progression of hepatocellular carcinoma. Int. J. Mol. Sci..

[42] Liu W., Cao Y., Wang T., Xiang G., Lu J., Zhang J., Hou P. (2013). The N-glycosylation modification of LHBs (large surface proteins of HBV) effects on endoplasmic reticulum stress, cell proliferation and its secretion. Hepat. Mon..

[43] Lazar C., Uta M., Branza-Nichita N. (2014). Modulation of the unfolded protein response by the human hepatitis B virus. Front. Microbiol..

[44] Kim S.Y., Kyaw Y.Y., Cheong J. (2017). Functional interaction of endoplasmic reticulum stress and hepatitis B virus in the pathogenesis of liver diseases. World J. Gastroenterol..

[45] Wang H.C., Huang W., Lai M.D., Su I.J. (2006). Hepatitis B virus pre-S mutants, endoplasmic reticulum stress and hepatocarcinogenesis. Cancer Sci..

[46] Wang H.C., Wu H.C., Chen C.F., Fausto N., Lei H.Y., Su I.J. (2003). Different types of ground glass hepatocytes in chronic hepatitis B virus infection contain specific pre-S mutants that may induce endoplasmic reticulum stress. Am. J. Pathol..

[47] Lazar C., Macovei A., Petrescu S., Branza-Nichita N. (2012). Activation of ERAD pathway by human hepatitis B virus modulates viral and subviral particle production. PLoS ONE.

[48] Tang H., Da L., Mao Y., Li Y., Li D., Xu Z., Li F., Wang Y., Tiollais P., Li T. (2009). Hepatitis B virus X protein sensitizes cells to starvation-induced autophagy via up-regulation of beclin 1 expression. Hepatology.

[49] Fan Y.F., Lu C.C., Chen W.C., Yao W.J., Wang H.C., Chang T.T., Lei H.Y., Shiau A.L., Su I.J. (2001). Prevalence and significance of hepatitis B virus (HBV) pre-S mutants in serum and liver at different replicative stages of chronic HBV infection. Hepatology.

[50] Su I.-J., Wang H.-C., Wu H.-C., Huang W. (2008). Ground glass hepatocytes contain pre-S mutants and represent preneoplastic lesions in chronic hepatitis B virus infection. J. Gastroenterol. Hepatol..

[51] Hsieh Y.H., Su I.J., Wang H.C., Chang W.W., Lei H.Y., Lai M.D., Chang W.T., Huang W. (2004). Pre-S mutant surface antigens in chronic hepatitis B virus infection induce oxidative stress and DNA damage. Carcinogenesis.

[52] Siddiquey M.N.A., Zhang H., Nguyen C.C., Domma A.J., Kamil J.P. (2018). The human cytomegalovirus endoplasmic reticulum-resident glycoprotein UL148 activates the unfolded protein response. J. Virol..

[53] Yao W., Cai H., Li X., Li T., Hu L., Peng T. (2014). Endoplasmic reticulum stress links hepatitis C virus RNA replication to wild-type PGC-1α/liver-specific PGC-1α upregulation. J. Virol..

[54] Alberts B., Johnson A., Lewis J., Raff M., Roberts K., Walter P. (2002). Molecular Biology of the Cell.

[55] Bravo R., Parra V., Gatica D., Rodriguez A.E., Torrealba N., Paredes F., Wang Z.V., Zorzano A., Hill J.A., Jaimovich E. (2013). Endoplasmic reticulum and the unfolded protein response: Dynamics and metabolic integration. Int. Rev. Cell Mol. Biol..

[56] Hetz C., Papa F.R. (2018). The unfolded protein response and cell fate control. Mol. Cell.

[57] Ruggiano A., Foresti O., Carvalho P. (2014). ER-associated degradation: Protein quality control and beyond. J. Cell Biol..

[58] Kadowaki H., Nishitoh H. (2013). Signaling pathways from the endoplasmic reticulum and their roles in disease. Genes.

[59] Hung J.H., Su I.J., Lei H.Y., Wang H.C., Lin W.C., Chang W.T., Huang W., Chang W.C., Chang Y.S., Chen C.C. (2004). Endoplasmic reticulum stress stimulates the expression of cyclooxygenase-2 through activation of NF-kappaB and pp38 mitogen-activated protein kinase. J. Biol. Chem..

[60] Liu H., Xu J., Zhou L., Yun X., Chen L., Wang S., Sun L., Wen Y., Gu J. (2011). Hepatitis B virus large surface antigen promotes liver carcinogenesis by activating the Src/PI3K/Akt pathway. Cancer Res..

[61] Tai P.-C., Suk F.M., Gerlich W., Neurath R., Shih C. (2002). Hypermodification and immune escape of an internally deleted middle envelope (M) protein of frequent and predominant hepatitis B virus variants. Virology.

[62] Chua P.K., Wang Y.L., Lin M.H., Masuda T., Suk F.M., Shih C. (2005). Reduced secretion of virions and hepatitis B virus (HBV) surface antigen of a naturally occurring HBV variant correlates with the accumulation of the small S envelope protein in the endoplasmic reticulum and Golgi apparatus. J. Virol..

[63] Schoeman J.C., Hou J., Harms A.C., Vreeken R.J., Berger R., Hankemeier T., Boonstra A. (2016). Metabolic characterization of the natural progression of chronic hepatitis B. Genome Med..

[64] Ghemrawi R., Battaglia-Hsu S.-F., Arnold C. (2018). Endoplasmic reticulum stress in metabolic disorders. Cells.

[65] Hung J.H., Yan C.W., Su I.J., Wang H.C., Lei H.Y., Lin W.C., Chang W.T., Huang W., Lu T.J., Lai M.D. (2010). Hepatitis B virus surface antigen interacts with acid alpha-glucosidase and alters glycogen metabolism. Hepatol. Res..

[66] Uemura A., Oku M., Mori K., Yoshida H. (2009). Unconventional splicing of XBP1 mRNA occurs in the cytoplasm during the mammalian unfolded protein response. J. Cell Sci..

[67] López-Hernández B., Ceña V., Posadas I. (2015). The endoplasmic reticulum stress and the HIF-1 signalling pathways are involved in the neuronal damage caused by chemical hypoxia. Br. J. Pharmacol..

[68] Gonen N., Meller A., Sabath N., Shalgi R. (2019). Amino acid biosynthesis regulation during endoplasmic reticulum sgtress is coupled to protein expression demands. iScience.

[69] Kasai S., Yamazaki H., Tanji K., Engler M.J., Matsumiya T., Itoh K. (2019). Role of the ISR-ATF4 pathway and its cross talk with Nrf2 in mitochondrial quality control. J. Clin. Biochem. Nutr..

[70] Yoshizawa T., Hinoi E., Jung D.Y., Kajimura D., Ferron M., Seo J., Graff J.M., Kim J.K., Karsenty G. (2009). The transcription factor ATF4 regulates glucose metabolism in mice through its expression in osteoblasts. J. Clin. Investig..

[71] Teng C.-F., Hsieh W.-C., Wu H.-C., Lin Y.-J., Tsai H.-W., Huang W., Su I.-J. (2015). Hepatitis B virus pre-S2 mutant induces aerobic glycolysis through mammalian target of rapamycin signal cascade. PLoS ONE.

[72] Teng C.-F., Wu H.-C., Tsai H.-W., Shiah H.-S., Huang W., Su I.-H. (2011). Novel feedback inhibition of surface antigen synthesis by mammalian target of rapamycin (mTOR) signal and its implication for hepatitis B virus tumorigenesis and therapy. Hepatology.

[73] van der Harg J.M., van Heest J.C., Bangel F.N., Patiwael S., van Weering J.R., Scheper W. (2017). The UPR reduces glucose metabolism via IRE1 signaling. Biochim. Biophys. Acta Mol. Cell Res..

[74] Amen O.M., Sarker S.D., Ghildyal R., Arya A. (2019). Endoplasmic reticulum stress activates unfolded protein response signaling and mediates inflammation, obesity, and cardiac dysfunction: Therapeutic and molecular approach. Front. Pharmacol..

[75] Oyadomari S., Harding H.P., Zhang Y., Oyadomari M., Ron D. (2008). Dephosphorylation of translation initiation factor 2alpha enhances glucose tolerance and attenuates hepatosteatosis in mice. Cell Metab..

[76] Chang Y.S., Tsai C.T., Huangfu C.A., Huang W., Lei H.Y., Lin C.F., Su I.J., Chang W.T., Wu P.H., Chen Y.T. (2011). ACSL3 and GSK-3β are essential for lipid upregulation induced by endoplasmic reticulum stress in liver cells. J. Cell Biochem..

[77] Teng C.F., Wu H.C., Hsieh W.C., Tsai H.W., Su I.J. (2015). Activation of ATP citrate lyase by mTOR signal induces disturbed lipid metabolism in hepatitis B virus pre-S2 mutant tumorigenesis. J. Virol..

[78] Stieger B. (2011). The role of the sodium-taurocholate cotransporting polypeptide (NTCP) and of the bile salt export pump (BSEP) in physiology and pathophysiology of bile formation. Handb. Exp. Pharmacol..

[79] Chiang J.Y.L. (2013). Bile acid metabolism and signaling. Compr. Physiol..

[80] Slijepcevic D., Kaufman C., Wichers C.G.K., Gilglioni E.H., Lempp F.A., Duijst S., de Waart D.R., Elferink R.P.J.O., Mier W., Stieger B. (2015). Impaired uptake of conjugated bile acids and hepatitis b virus pres1-binding in na(+) -taurocholate cotransporting polypeptide knockout mice. Hepatology.

[81] Blank A., Eidam A., Haag M., Hohmann N., Burhenne J., Schwab M., de Graaf S.V., Meyer M.R., Maurer H.H., Meier K. (2018). The NTCP-inhibitor Myrcludex B: Effects on bile acid disposition and tenofovir pharmacokinetics. Clin. Pharmacol. Ther..

[82] Osiowy C., Giles E., Tanaka Y., Mizokami M., Minuk G.Y. (2006). Molecular evolution of hepatitis B virus over 25 years. J. Virol..

[83] Clark D.N., Hu J. (2015). Unveiling the roles of HBV polymerase for new antiviral strategies. Future Virol..

[84] Coffin C.S., Mulrooney-Cousins P.M., Peters M.G., van Marle G., Roberts J.P., Michalak T.I., Terrault N.A. (2011). Molecular characterization of intrahepatic and extrahepatic hepatitis B virus (HBV) reservoirs in patients on suppressive antiviral therapy. J. Viral Hepat..

[85] Zhang Z.-H., Wu C.-C., Chen X.-W., Li X., Li J., Lu M.-J. (2016). Genetic variation of hepatitis B virus and its significance for pathogenesis. World J. Gastroenterol..

[86] Kim H., Lee S.-A., Do S.Y., Kim B.-J. (2016). Precore/core region mutations of hepatitis B virus related to clinical severity. World J. Gastroenterol..

[87] Hsieh Y.H., Su I.J., Yen C.J., Liu Y.R., Liu R.J., Hsieh W.C., Tsai H.W., Wang L.H.C., Hsu C.C., Huang W. (2015). Hepatitis B virus pre-S_2_ mutant surface protein inhibits DNA double strand break repair and leads to genome instability in hepatitis B virus hepatocarcinogenesis. J. Pathol..

[88] Hsieh Y.H., Su I.J., Wang H.C., Tsai J.H., Huang Y.J., Chang W.W., Lai M.D., Lei H.Y., Huang W. (2007). Hepatitis B virus pre-S2 mutant surface antigen induces degradation of cyclin-dependent kinase inhibitor p27Kip1 through c-Jun activation domain-binding protein 1. Mol. Cancer Res..

[89] Chen B.-F., Liu C.-J., Jow G.-M., Cheng P.-J., Kao J.-H., Chen D.-S. (2006). High prevalence and mapping of pre-S deletion in hepatitis B virus carriers with progressive liver diseases. Gastroenterology.

[90] Hadziyannis S., Gerber M.A., Vissoulis C., Popper H. (1973). Cytoplasmic hepatitis B antigen in “ground-glass” hepatocytes of carriers. Arch. Pathol..

[91] Callea F., De Vos R., Togni R., Tardanico R., Vanstapel M.J., Desmet V.J. (1986). Fibrinogen inclusions in liver cells: A new type of ground-glass hepatocyte. Immune light and electron microscopic characterization. Histopathology.

[92] Fan Y.F., Lu C.C., Chang Y.C., Lin P.W., Su I.J. (2000). Identification of a pre-S2 mutant in hepatocytes expressing a novel marginal pattern of surface antigen in advanced diseases of chronic hepatitis B virus infection. J. Gastroenterol. Hepatol..

[93] Prange R., Streeck R.E. (1995). Novel transmembrane topology of the hepatitis B virus envelope proteins. EMBO J..

[94] Shen F.-C., Su I.-J., Wu H.-C., Hsieh Y.-H., Yao W.-J., Young K.-C., Chang T.-C., Hsieh H.-C., Tsai H.-N., Huang W. (2009). A Pre-S Gene Chip to detect the pre-S deletions in the hepatitis B virus large surface antigen as a predictive marker for hepatoma risk in the chronic HBV carriers. J. Biomed. Sci..

[95] Wang H.-C., Chang W.-T., Chang W.-W., Wu H.-C., Huang W., Lei H.-Y., Lai M.-D., Fausto N., Su I.-J. (2005). Hepatitis B virus pre-S2 mutant upregulates cyclin A expression and induces nodular proliferation of hepatocytes. Hepatology.

[96] Yang J.-C., Teng C.-F., Wu H.-C., Tsai H.-W., Chuang H.-C., Tsai T.-F., Hsu Y.-H., Huang W., Wu L.-W., Su I.-J. (2009). Enhanced expression of vascular endothelial growth factor-A in ground glass hepatocytes and its implication in hepatitis B virus hepatocarcinogenesis. Hepatology.

[97] Viganò M., Lampertico P. (2012). Clinical implications of HBsAg quantification in patients with chronic hepatitis B. Saudi J. Gastroenterol..

[98] Pfefferkorn M., Böhm S., Schott T., Deichsel D., Bremer C.M., Schröder K., Gerlich W.H., Glebe D., Berg T., van Bömmel F. (2018). Quantification of large and middle proteins of hepatitis B virus surface antigen (HBsAg) as a novel tool for the identification of inactive HBV carriers. Gut.

[99] Patient R., Hourioux C., Sizaret P.-Y., Trassard S., Sureau C., Roingeard P. (2007). Hepatitis B virus subviral envelope particle morphogenesis and intracellular trafficking. J. Virol..

[100] Cao J., Zhang J., Lu Y., Luo S., Zhang J., Zhu P. (2019). Cryo-EM structure of native spherical subviral particles isolated from HBV carriers. Virus Res..

[101] Bruss V., Gerhardt E., Vieluf K., Wunderlich G. (1996). Functions of the large hepatitis B virus surface protein in viral particle morphogenesis. Intervirology.

[102] Raney A.K., Easton A.J., Milich D.R., McLachlan A. (1991). Promoter-specific transactivation of hepatitis B virus transcription by a glutamine- and proline-rich domain of hepatocyte nuclear factor 1. J. Virol..

[103] Hildt E., Hofschneider P.H. (1998). The PreS2 activators of the hepatitis B virus: Activators of tumour promoter pathways. Recent Res. Cancer Res..

[104] Chen L., Hou J., Wei H.-S., Liu A.-X., Li P.-R., Zhao J., Liu J., Li B.-A. (2017). Characterization and clinical application of a monoclonal antibody to hepatitis B virus large surface proteins. Front. Lab. Med..

[105] Zhu X., Gong Q., Yu D., Zhang D., Gu L., Han Y., Chen J., Zhang Y., Zhang X. (2013). Early serum hepatitis B virus large surface protein level: A stronger predictor of virological response to peginterferon alfa-2a than that to entecavir in HBeAg-positive patients with chronic hepatitis B. J. Clin. Virol..

[106] Yen C.J., Ai Y.L., Tsai H.W., Chan S.H., Yen C.S., Cheng K.H., Lee Y.P., Kao C.W., Wang Y.C., Chen Y.L. (2018). Hepatitis B virus surface gene pre-S_2_ mutant as a high-risk serum marker for hepatoma recurrence after curative hepatic resection. Hepatology.

